# Construction of an immune-related lncRNA signature pair for predicting oncologic outcomes and the sensitivity of immunosuppressor in treatment of lung adenocarcinoma

**DOI:** 10.1186/s12931-022-02043-4

**Published:** 2022-05-13

**Authors:** Jinman Zhuang, Zhongwu Chen, Zishan Chen, Jin Chen, Maolin Liu, Xinying Xu, Yuhang Liu, Shuyan Yang, Zhijian Hu, Fei He

**Affiliations:** 1 Department of Epidemiology and Health Statistics, School of Public Health, Fujian Medical University, Fuzhou, China; 2 Fujian Provincial Key Laboratory of Tumor Microbiology, Fujian Medical University, Fuzhou, China; 3 Fujian Digital Tumor Data Research Center, Fuzhou, China; 4 Department of Interventional Therapy, The First Affiliated Hospital of Fujian Medical University, Fuzhou, China

**Keywords:** LUAD, IRLPs signature, Biomarker, Prognosis, Immunosuppressant

## Abstract

**Background:**

Although immunotherapy has shown clinical activity in lung adenocarcinoma (LUAD), LUAD prognosis has been a perplexing problem. We aimed to construct an immune-related lncRNA pairs (IRLPs) score for LUAD and identify what immunosuppressor are appropriate for which group of people with LUAD.

**Methods:**

Based on The Cancer Genome Atlas (TCGA)-LUAD cohort, IRLPs were identified to construct an IRLPs scoring system by Cox regression and validated in the Gene Expression Omnibus (GEO) dataset using log-rank test and the receiver operating characteristic curve (ROC). Next, we used spearman’s correlation analysis, *t*-test, signaling pathways analysis and gene mutation analysis to explore immune and molecular characteristics in different IRLP subgroups. The “pRRophetic” package was used to predict the sensitivity of immunosuppressant.

**Results:**

The IRLPs score was constructed based on eight IRLPs calculated as 2.12 × (MIR31HG|RRN3P2) + 0.43 × (NKX2-1-AS1|AC083949.1) + 1.79 × (TMPO-AS1|LPP-AS2) + 1.60 × (TMPO-AS1|MGC32805) + 1.79 × (TMPO-AS1|PINK1-AS) + 0.65 × (SH3BP5-AS1|LINC01137) + 0.51 × (LINC01004|SH3PXD2A-AS1) + 0.62 × (LINC00339|AGAP2-AS1). Patients with a lower IRLPs risk score had a better overall survival (OS) (Log-rank test *P*_TCGA train dataset_ < 0.001, *P*_TCGA test dataset_ = 0.017, *P*_GEO dataset_ = 0.027) and similar results were observed in the AUCs of TCGA dataset and GEO dataset (AUC _TCGA train dataset_ = 0.777, AUC _TCGA test dataset_ = 0.685, AUC _TCGA total dataset_ = 0.733, AUC _GEO dataset_ = 0.680). Immune score (Cor = -0.18893, *P* < 0.001), stoma score (Cor = -0.24804, *P* < 0.001), and microenvironment score (Cor = -0.22338, *P* < 0.001) were significantly decreased in the patients with the higher IRLP risk score. The gene set enrichment analysis found that high-risk group enriched in molecular changes in DNA and chromosomes signaling pathways, and in this group the tumor mutation burden (TMB) was higher than in the low-risk group (*P* = 0.0015). Immunosuppressor methotrexate sensitivity was higher in the high-risk group (*P* = 0.0052), whereas parthenolide (*P* < 0.001) and rapamycin (*P* = 0.013) sensitivity were lower in the high-risk group.

**Conclusions:**

Our study established an IRLPs scoring system as a biomarker to help in the prognosis, the identification of molecular and immune characteristics, and the patient-tailored selection of the most suitable immunosuppressor for LUAD therapy.

**Supplementary Information:**

The online version contains supplementary material available at 10.1186/s12931-022-02043-4.

## Background

Lung cancer is one of the most common malignant tumors globally, with a high incidence of 11.4% in 2020. Approximately 40% of the primary lung tumors are lung adenocarcinomas (LUAD) [1, 2]. LUAD, which is common in females and non-smokers, is characterized by high mortality and metastasis rates [3]. Although great improvement has been made in the clinical diagnosis and treatment, the 5-year survival rate in LUAD patients is only 18% [4]. Therefore, the identification of new biomarkers to help in the prognosis of LUAD is of great significance.

Chemotherapeutic is one of the most effective ways in the treatment of advanced non-small cell lung cancer. However, drug resistance is a is a major problem that puzzles researchers. Immunosuppressants recently were found to be potential anti-cancer drugs in LUAD. Previous studies have shown that rapamycin and parthenolide will result some beneficial modulation in lung cancer chemotherapy [5–7]. Recently, the emergence of immunotherapy has brought unprecedented levels of survival to lung cancer patients, especially those with advanced or metastatic LUAD [8, 9]. However, immunotherapy brings not only considerable therapeutic effects but also immune-related adverse events (ir AEs). Corticosteroid therapy can successfully treat most ir AEs, but a combination of immunosuppressors is needed to combat more serious adverse reactions [10, 11]. Immunosuppressant methotrexate were used to treat rheumatic ir AEs [11], and high-dose methotrexate can be applied to immunochemotherapy in many type of cancers [12, 13]. However, since it is unclear whether a patient can undergo immunosuppressive therapy of these immunosuppressors safely, there is an urgent need to find some biomarkers to predict the drug sensitivity of immunosuppressants.

LUAD is an immune-sensitive cancer, studies have shown that the immunotherapy response may be predicted by tumor-immune cell infiltration and an immune score[14]. Long non-coding RNAs (lncRNAs) are RNAs without protein-coding capacity and greater than 200 nucleotides in length[15]. Studies showed that lncRNAs can regulate the immune response and immune cell development[16, 17]. Several studies have proposed immune-related lncRNA signatures to help in the prognostic of LUAD. However, the results cannot be directly generalized to all patients due to the use of different chip sequencing protocols, different platforms, and different testing times for gene expression [18–20]. These shortcomings could be overcome by combining two or more biomarkers, which work better than a single prediction criterion in cancer prediction models, and immune-related gene pairs (IRLPs) were reported to have accurately predicted the LUAD prognoses [21, 22]. However, these studies have focused on mRNAs rather than lncRNAs, which play an important role in the immune system. Therefore, the clinical relevance and prognostic significance of immune-related lncRNAs pairs (IRLPs) are currently unclear.

In this study, we constructed an individualized signature of IRLPs that works as an independent and predictive factor of overall survival (OS) for LUAD patients. Furthermore, the IRLP model also helps distinguishing the LUAD patients responsive to immunotherapy and predicts the sensitivity of immunosuppressors used in treatment of LUAD.

## Materials and methods

### Data source

The RNA-seq data of 515 LUAD cases (including 535 tumor samples and 59 normal samples) and 569 LUAD cases of genetic alteration data were downloaded from The Cancer Genome Atlas (TCGA)-LUAD cohort (https://portal.gdc.cancer.gov/) (October 10th, 2020). In addition, the normalized data of RNA expression matrix of GSE30219, GSE37745, and GSE50081 (we selected the RNA expression datasets which were normalized and measured from the same platform after searched LUAD RNA expression data) were downloaded from Gene Expression Omnibus (GEO https://www.ncbi.nlm.nih.gov/geo/); the platform of these datasets was GPL570 (Affymetrix Human Genome U133 Plus 2.0 Array). The relevant clinical characteristics of patients were also downloaded, and the patients without the information on survival time and survival status were excluded from our study.

### Identification of immune-related lncRNAs

Two thousand four hundred ninety-eight immune-related genes were downloaded from the ImmPort Portal (https://www.immport.org/). Then, immune-related lncRNAs were identified by Pearson’s correlation analysis between immune-related genes and lncRNA expression levels (|*correlation coefficient*|> 0.6 and *p* < 0.001).

### Construction of a prognostic IRLP signature

To ensure that the immune-related lncRNAs could be measured on all platforms included in this study, the intersect function was used to identify the common immune-related lncRNAs in the TCGA and GEO datasets. We only selected the lncRNAs with a relatively high variation in expression levels (median absolute deviation > 0.5). Next, the immune-related lncRNAs were paired randomly to construct a collection of lncRNA pairs. For each LUAD sample, the IRLPs were computed by pairwise comparison of the expression level. The output is one if the expression of the first lncRNA is higher than that of the second one; otherwise, the output is zero. We screened out overlapping IRLPs in TCGA and GEO dataset, after removing IRLPs with small variation and imbalanced distribution (MAD = 0), the remaining ones were selected as candidate IRLPs. The TCGA dataset was randomly divide into train dataset and test dataset and we performed univariate Cox regression analysis and LASSO regression analysis with tenfold cross-validation to find out OS-related IRLPs in TCGA train dataset. Finally, multivariate Cox regression analysis was carried out to identify top OS-related IRLPs and to establish the final model of an IRLP risk score to predict the prognosis of LUAD. The IRLP risk score was calculated using the following formula:

$$\mathrm{IRLP risk score}=\sum_{\mathrm{i}=1}^{\mathrm{n}}{\mathrm{coef}}_{\mathrm{i}}\times {\mathrm{x}}_{\mathrm{i}}$$

where coef_i_ is the coefficient and x_i_ is the output of pairwise comparison of expression level in each sample. (methods in selection of 8 IRLPs step by step were shown in the supplement).

### Validation of IRLPs signature in the GEO data set

The IRLP model was further evaluated in the LUAD patients from the GEO dataset by the log-rank test. We also accessed the prognosis value of the IRLP risk score based on other clinical factors in univariate and multivariate Cox regression analysis. The receiver operating characteristic curve (ROC) was used to evaluate the predicting accuracy of this signature by calculating the area under ROC (AUC).

### Comprehensive analysis of immune characteristic and molecular variation in different IRLP risk score subgroups

Data on the infiltration of immune cells found in the TCGA dataset were downloaded from TIMER2.0 (http://timer.comp-genomics.org), a website that provides four modules for investigating the associations between immune infiltrates and genetic or clinical features, and four modules for exploring cancer-related associations in the TCGA cohorts [23]. Spearman’s correlation analysis was performed to analyze the relationship between the immune cell infiltrates and the IRLPs risk score. In addition, Student’s *t*-test was used to compare the different levels of immune cell infiltrates between the high-risk and low-risk groups defined by the IRLP risk score.

Differential expression analysis was performed on all genes between the high-risk group and low-risk group of TCGA samples. In addition, gene set enrichment analysis was used to determine the signaling pathways based on the Kyoto Encyclopedia of Genes and Genomes (KEGG) and Gene Ontology (GO) gene set (GSEA software).

In the gene mutation analysis, gene mutation quantity and quality were analyzed in two subgroups of LUAD patients (Maftools package). In addition, we also analyzed the relationship between tumor mutation burden (TMB) and IPLPs risk score subgroup using a Student’s *t*-test.

### Predicting the drug sensitivity of the immunosuppressors

pRRophetic is an R package used to predict clinical chemotherapeutic response from tumor gene expression level, a ridge regression model was applied. Genes (ruled out genes with very low variability across samples) as predictors and the drug sensitivity (IC50) values (of the drug of interest) as the outcome variable[24]. pRRophetic package included prediction of drug sensitivity in immunosuppressants (methotrexate, parthenolide, rapamycin). In order to identify which immunosuppressant might be useful, we used the “pRRophetic” R package to predict drug sensitivity from tumor gene expression levels.

### Statistical analysis

All statistical analyses were performed in the R 4.0.5 software. Student’s *t*-test was used to compare the differences between two subgroups. The Kaplan–Meier method was used to analyze the differences in survival curves using the log-rank test.

## Results

### Dataset of LUAD patients

**Fig. 1:**
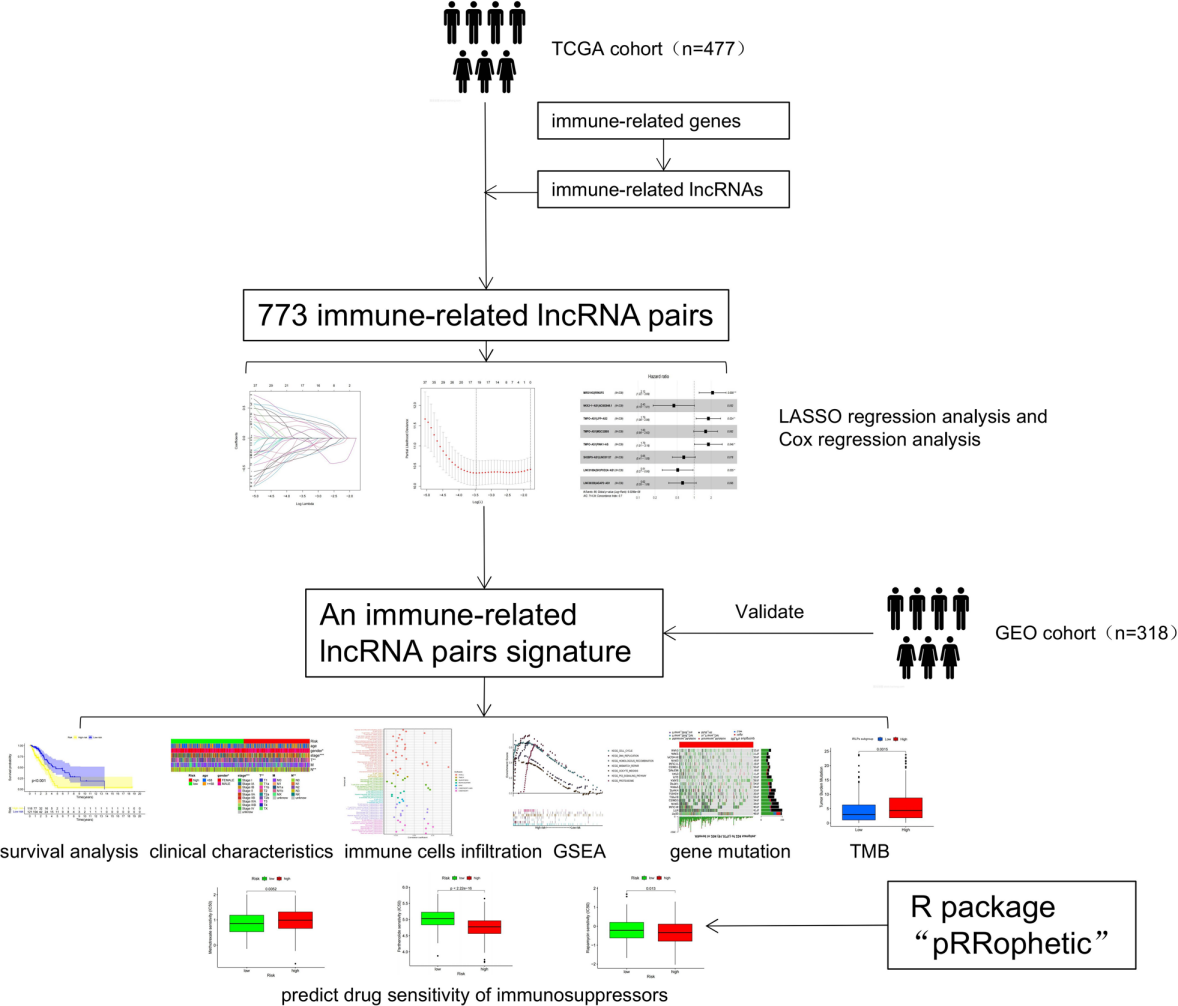
The flow diagram of this study

After excluding the patients for whom the survival status and survival time were missing, a total of 795 patients (TCGA-LUAD: 477 cases; GEO: 318 cases) were included in our study. All clinical characteristics (age, gender, stage, TNM grade) of TCGA and GEO dataset were present at Table 1. The flow diagram of this study was shown in Fig. 1.

**Table 1:** Clinical characteristics of TCGA and GEO dataset

Variable	TCGA-LUAD dataset (N = 477) N(%)	GEO LUAD dataset (N = 318)		
		GSE30219 N(%)	GSE37745 N(%)	GSE50081 N(%)
Age				
< 68	261 (54.7)	61 (73.5)	71 (67.0)	48 (37.2)
≥68	216 (45.3)	22 (26.5)	35 (33.0)	81 (62.8)
Gender				
Female	257 (53.9)	18 (21.7)	60 (56.6)	62 (48.1)
Male	220 (46.1)	65 (78.3)	46 (43.4)	67 (51.9)
Stage				
I	4 (0.8)	NA	NA	NA
IA	124 (26.0)	NA	NA	37 (28.7)
IB	125 (26.2)	NA	NA	56 (43.4)
II	191 (40.0)	NA	NA	NA
IIA				7 (5.4)
IIB				29 (22.5)
III	NA	NA	NA	0 (0)
IV	25 (5.2)	NA	NA	0 (0)
Unknow	8 (1.7)	NA	NA	0 (0)
T				
T_1_	159 (33.3)	69 (83.1)	NA	44 (34.1)
T_2_	254 (53.2)	12 (14.5)	NA	83 (64.3)
T_3_	43 (9.0)	2 (2.4)	NA	2 (1.6)
T_4_	18 (3.8)	0 (0)	NA	0 (0)
T_X_	3 (0.6)	0 (0)	NA	0 (0)
N				
N_0_	307 (64.4)	80 (96.4)	NA	95 (73.6)
N_1_	90 (18.9)	3 (3.6)	NA	34 (26.4)
N_2_	67 (14.0)	0 (0)	NA	0 (0)
N_X_	2 (0.4)	0 (0)	NA	0 (0)
Unknow	1 (0.2)	0 (0)	NA	0 (0)
M				
M_0_	313 (65.6)	83 (100)	NA	129 (100)
M_1_	24 (5.0)	0 (0)	NA	0 (0)
M_X_	136 (28.5)	0 (0)	NA	0 (0)

### Construction and validation of a prognostic IRLP signature

**Fig. 2:**
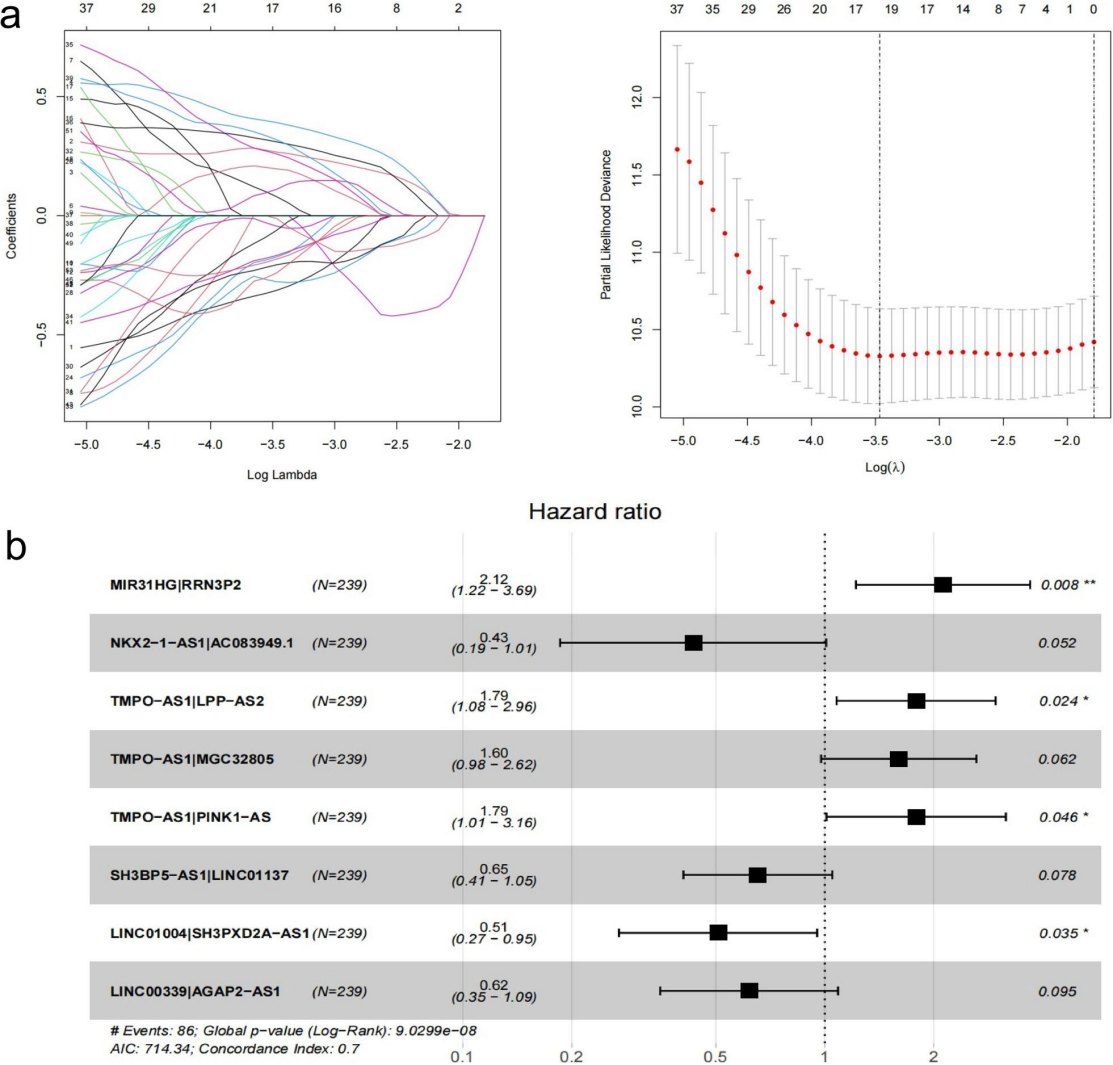
Construction of a IRLPs signature in the TCGA train set. **a** “Leaveone-out-cross-validation” for parameter selection in LASSO regression to flter out 18 IRLPs. **b** The forest map of multivariate Cox regression analysis to establish a IRLPs signature with 8 IRLPs in TCGA train dataset

**Fig. 3:**
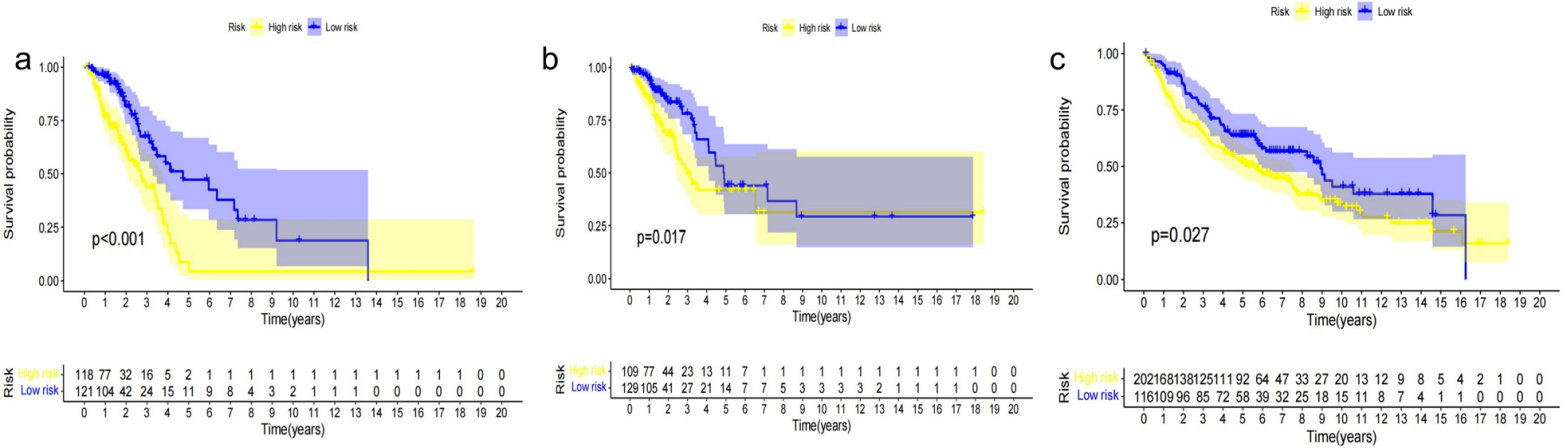
Kaplan–Meier survival curves of LUAD in IRLPs high-risk and low-risk group **a**) in the TCGA train dataset, **b** in the TCGA test dataset. **c** in the GEO dataset

A total of 105 immune-related lncRNAs were found in all platforms of the dataset, and 773 IRLPs were paired. First, univariate Cox regression identified 53 IRLPs that were related to the OS of LUAD patients in the TCGA train dataset (*P* < 0.01). Then, the Least Absolute Shrinkage and Selection Operator (LASSO) regression analysis with iteration = 1000 selected 18 IRLPs (Fig. 2a) for the multivariate Cox regression analysis, and, finally, eight IRLPs were identified to calculate the IRLP risk score (Fig. 2b). The IRLPs risk score was calculated as 2.12 × (MIR31HG|RRN3P2) + 0.43 × (NKX2-1-AS1|AC083949.1) + 1.79 × (TMPO-AS1|LPP-AS2) + 1.60 × (TMPO-AS1|MGC32805) + 1.79 × (TMPO-AS1|PINK1-AS) + 0.65 × (SH3BP5-AS1|LINC01137) + 0.51 × (LINC01004|SH3PXD2A-AS1) + 0.62 × (LINC00339|AGAP2-AS1). Furthermore, we compared the survival curves of the TCGA train dataset (*P* < 0.001), TCGA test dataset (*P* = 0.017), and GEO dataset (*P* = 0.027) (Fig. 3a–c). These results all showed that high-risk LUAD patients exhibited a poorer prognosis than low-risk LUAD patients.

### Assessing the value of the IRLP signature to predict the prognostic of overall survival

**Fig. 4:**
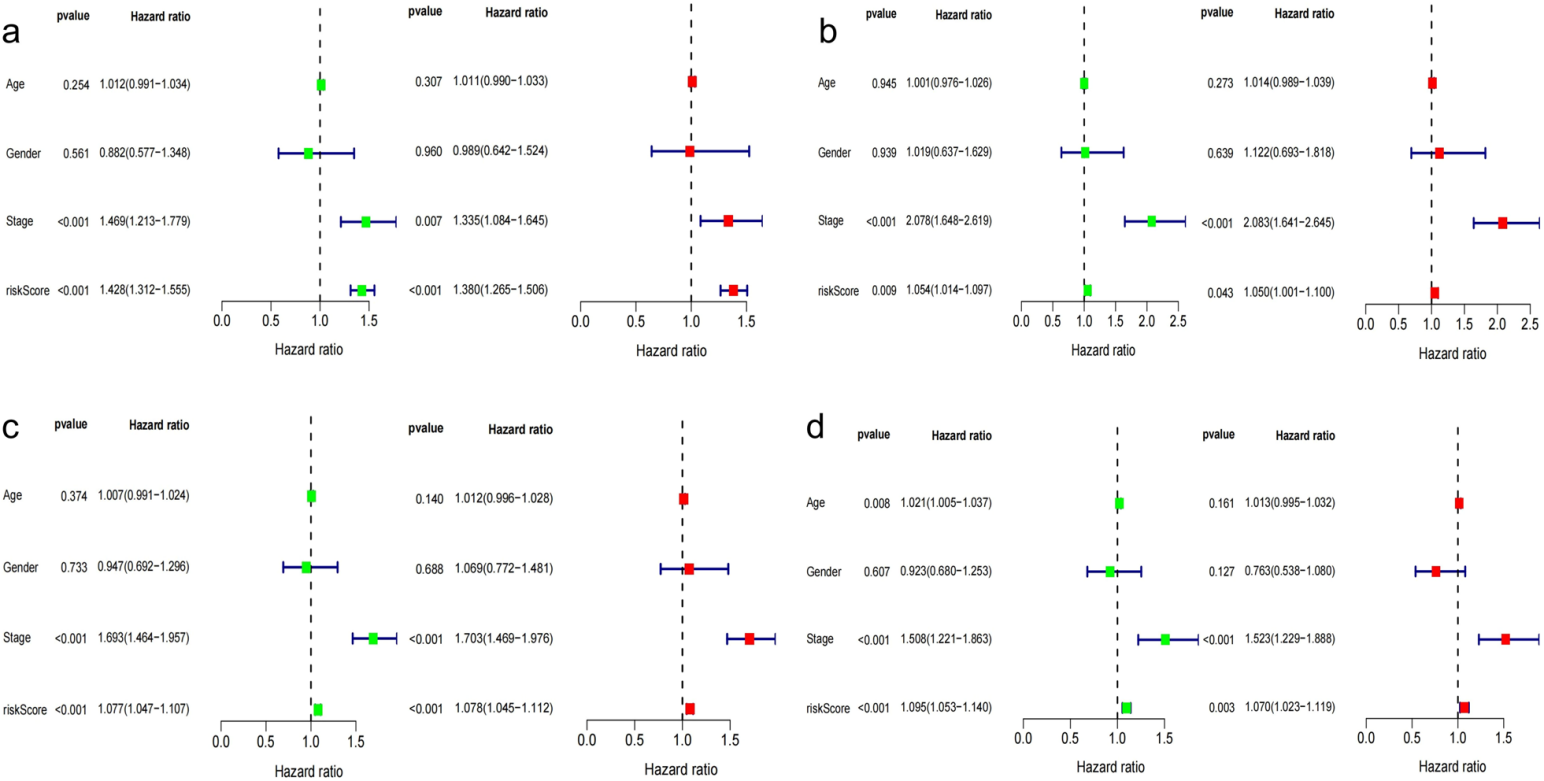
The forest map of univariate and multivariate Cox regression analysis of IRLPs risk score and clinical characteristics for the prognosis of LUAD patients. **a** in the TCGA train dataset, **b** in the TCGA test dataset. **c** in the TCGA total dataset, **d** in the GEO dataset

**Fig. 5:**
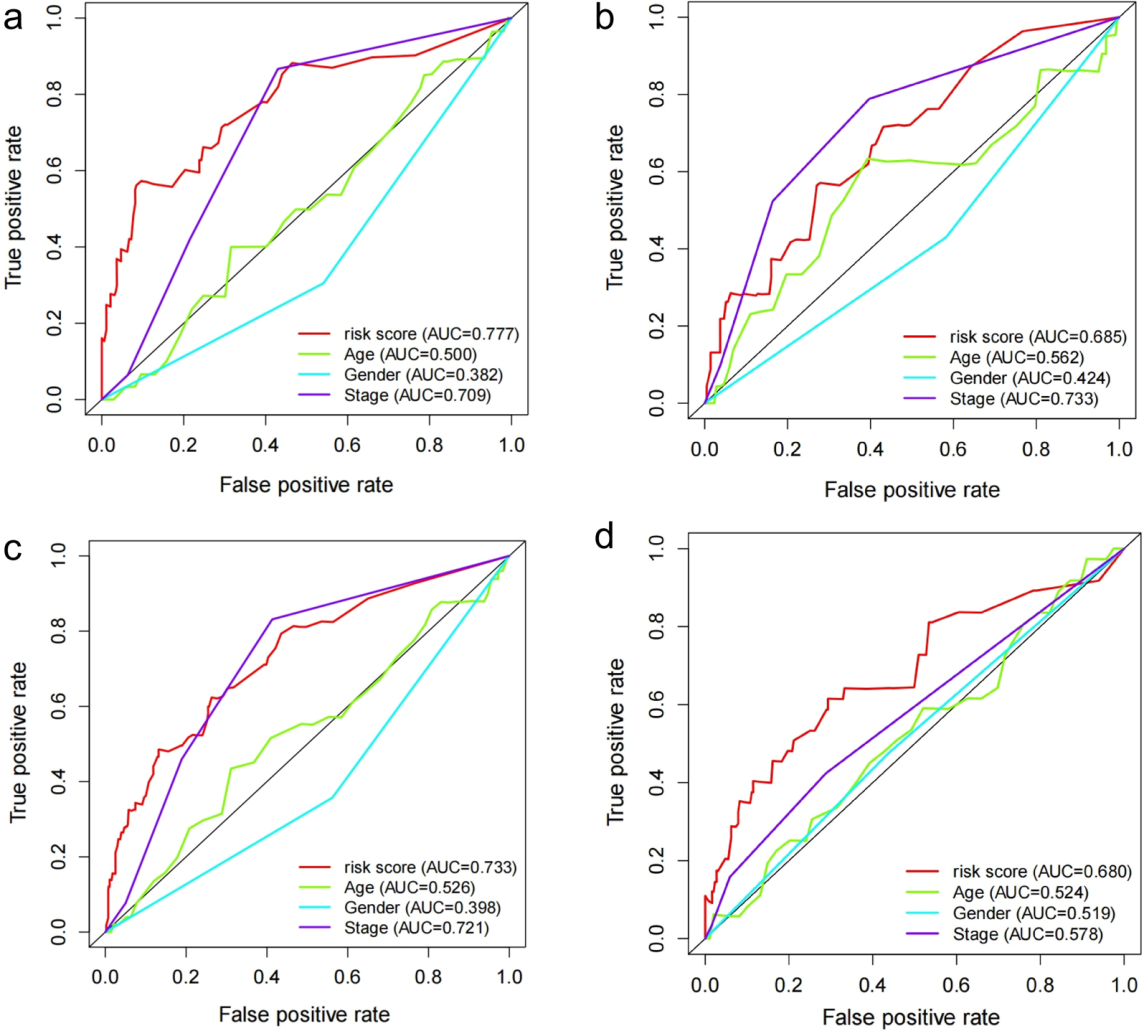
The ROC curves of the IRLPs risk score and other clinical characters **a** in the TCGA train dataset, **b** in the TCGA test dataset. (c) in the TCGA total dataset, **d** in the GEO dataset

We took advantage of the univariate and multivariate Cox regression model to analyze the predictive value of IRLP risk score and clinical parameters. The univariable Cox regression analysis indicated that the IRLPs risk score was an important factor for patients’ prognosis (TCGA total dataset: *HR* = 1.077 (95% *CI*: 1.047–1.107), *P* < 0.001; TCGA train dataset: *HR* = 1.428 (95% *CI*: 1.312–1.555), *P* < 0.001; TCGA test dataset: *HR* = 1.054 (95% *CI*: 1.014–1.097), *P* = 0.009; GEO dataset: *HR* = 1.095 (95% *CI*: 1.053–1.140), *P* < 0.001; Fig. 4a–d). The multivariable Cox regression indicated that the IRLPs risk score was an independent predictive indicator for the OS of LUAD patients (TCGA total dataset: *HR* = 1.078 (95% *CI* = 1.045–1.112), *P* < 0.001; TCGA train dataset: *HR* = 1.380 (95% *CI* = 1.265–1.507), *P* < 0.001; TCGA test dataset: *HR* = 1.050 (95% *CI* = 1.001–1.100), *P* = 0.043; GEO dataset: *HR* = 1.070 (95% *CI* = 1.023–1.119), *P* = 0.003; Fig. 4a–d). The ROC curves also revealed that the IRLPs risk score plays an important role in predicting LUAD prognosis (TCGA total dataset: AUC = 0.733; TCGA train dataset: AUC = 0.777; TCGA test dataset: AUC = 0.685; GEO dataset: AUC = 0.680 Fig. 5a–d).

### Relationship between the IRLP subgroups and clinical characteristics

**Fig. 6:**
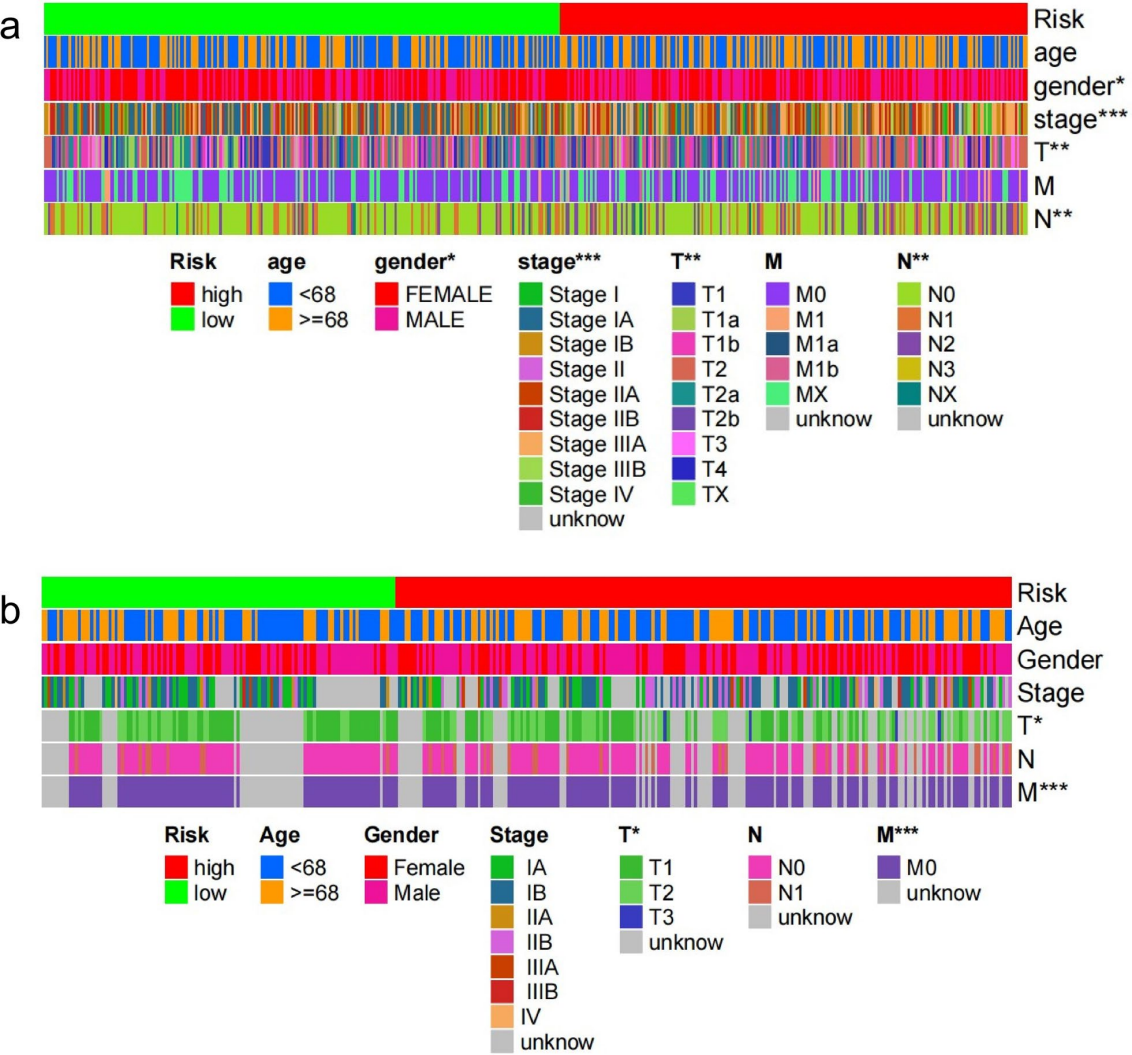
Heat map for relationship between IRLPs subgroups and clinical characters * means *P* < 0.05; ** means *P* < 0.01; *** means *P* < 0.001 **a** in the TCGA total dataset. **b** in the GEO dataset

We evaluated the correlation between the IRLP subgroups and clinical characteristics by heatmap. The results showed that the distributions of gender, stage, T stage, and N stage were significantly different between the high-risk and low-risk groups of the TCGA total dataset (Fig. 6a), and the GEO dataset showed that the distributions of the T and M stage were significantly different (Fig. 6b).

### Relationship between the IRLPs and immune cell infiltrates

**Fig. 7:**
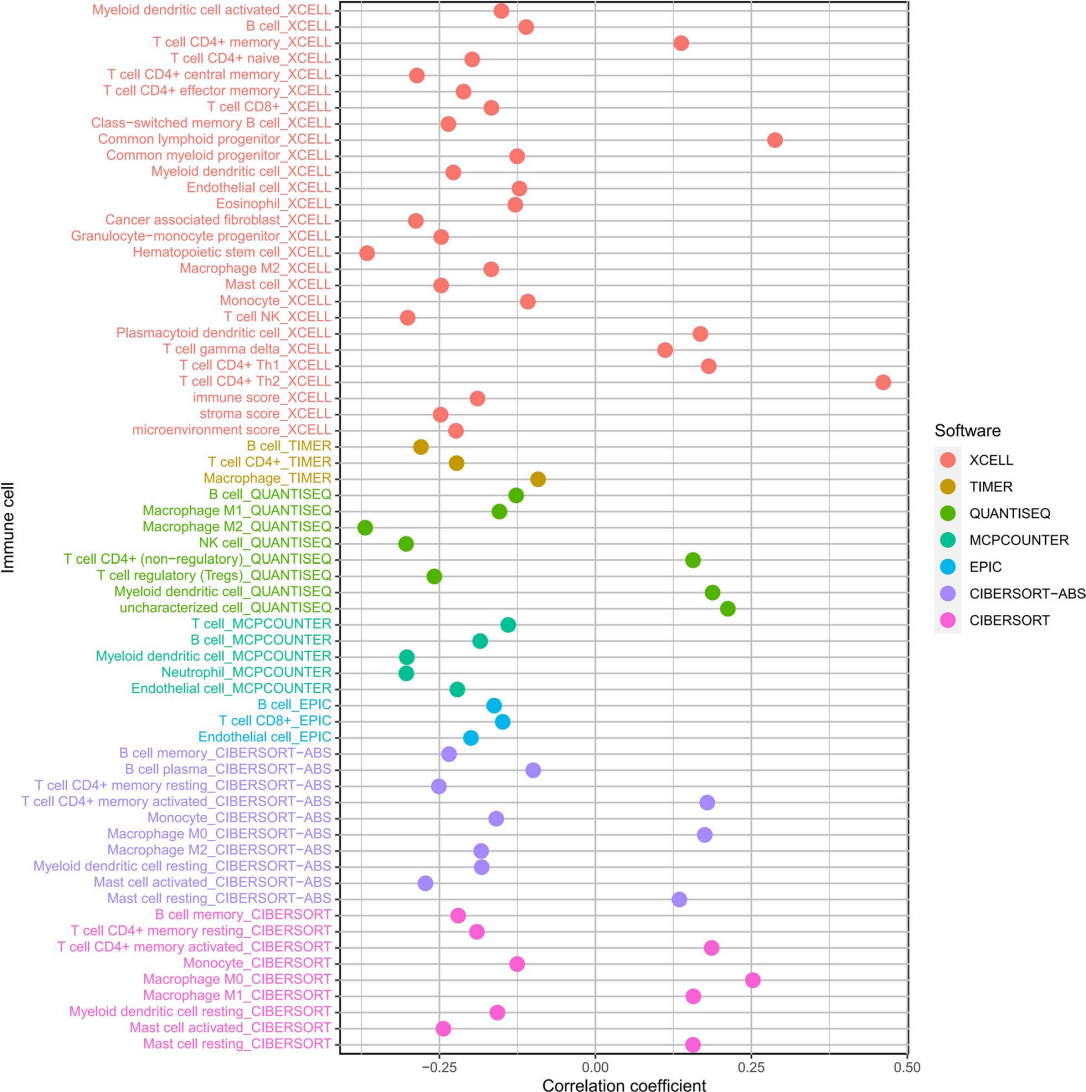
Relationship between the IRLPs risk score and immune cells infiltration

**Fig. 8:**
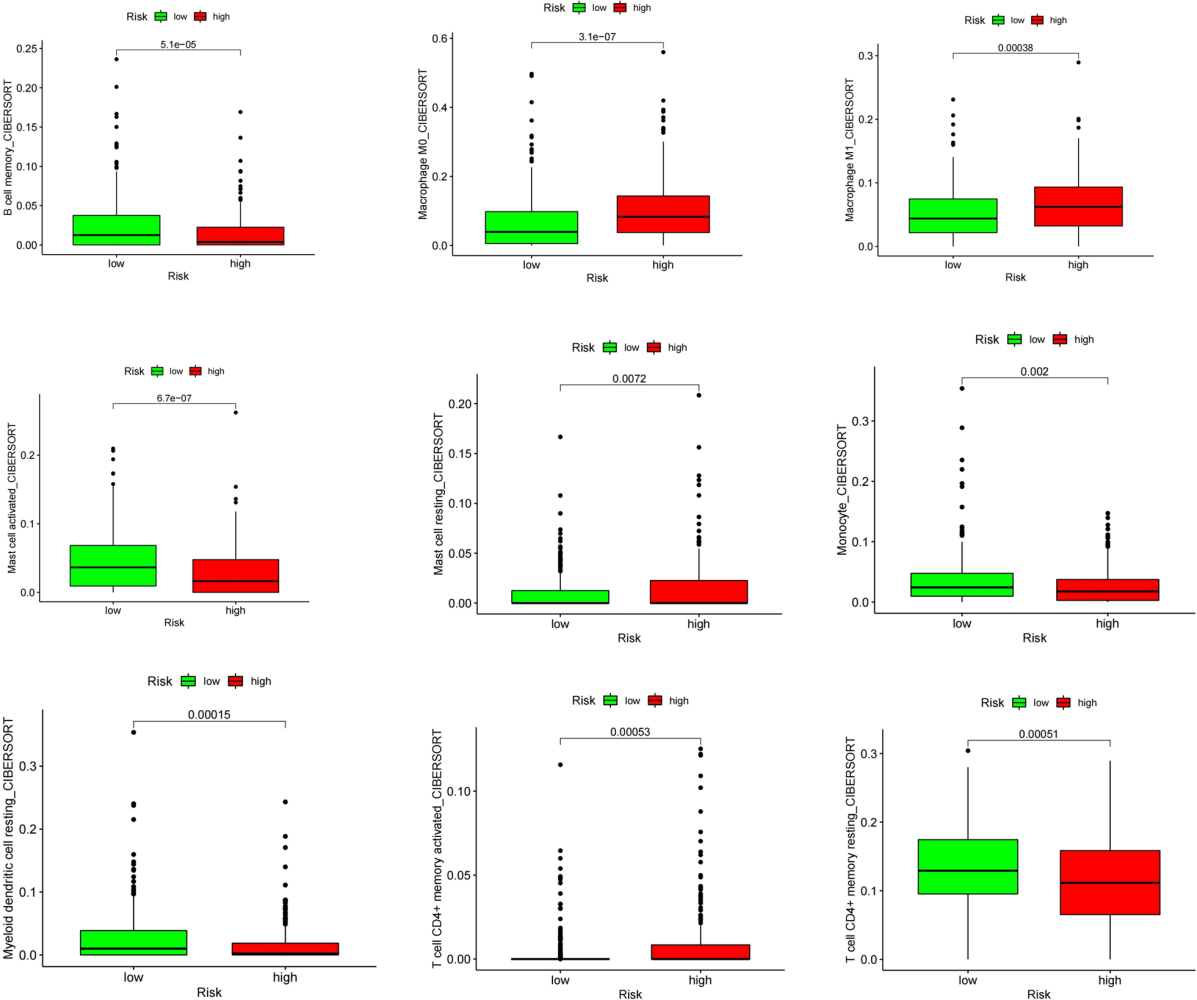
Relationship between the IRLPs subgroups and immune cells infiltration of CIBERSORT

Immune score reflect immune cells infiltrate situation, and stoma score reflect the stromal cells infiltrate. While microenvironment score reflect a a comprehensive situation of the former two scores, represents the overall picture of the immune microenvironment. Spearman’s rank correlation analysis showed that the immune score (Cor = − 0.18893, *P* < 0.001), stoma score (Cor = − 0.24804, *P* < 0.001), and microenvironment score (Cor = − 0.22338, *P* < 0.001) were significantly decreased in the group with the higher IRLP risk score (Fig. 7). The distributions of immune cell infiltrates were different in the two IRLP subgroups (Fig. 8).

### Molecular characteristics of different IRLP subgroups

**Fig. 9:**
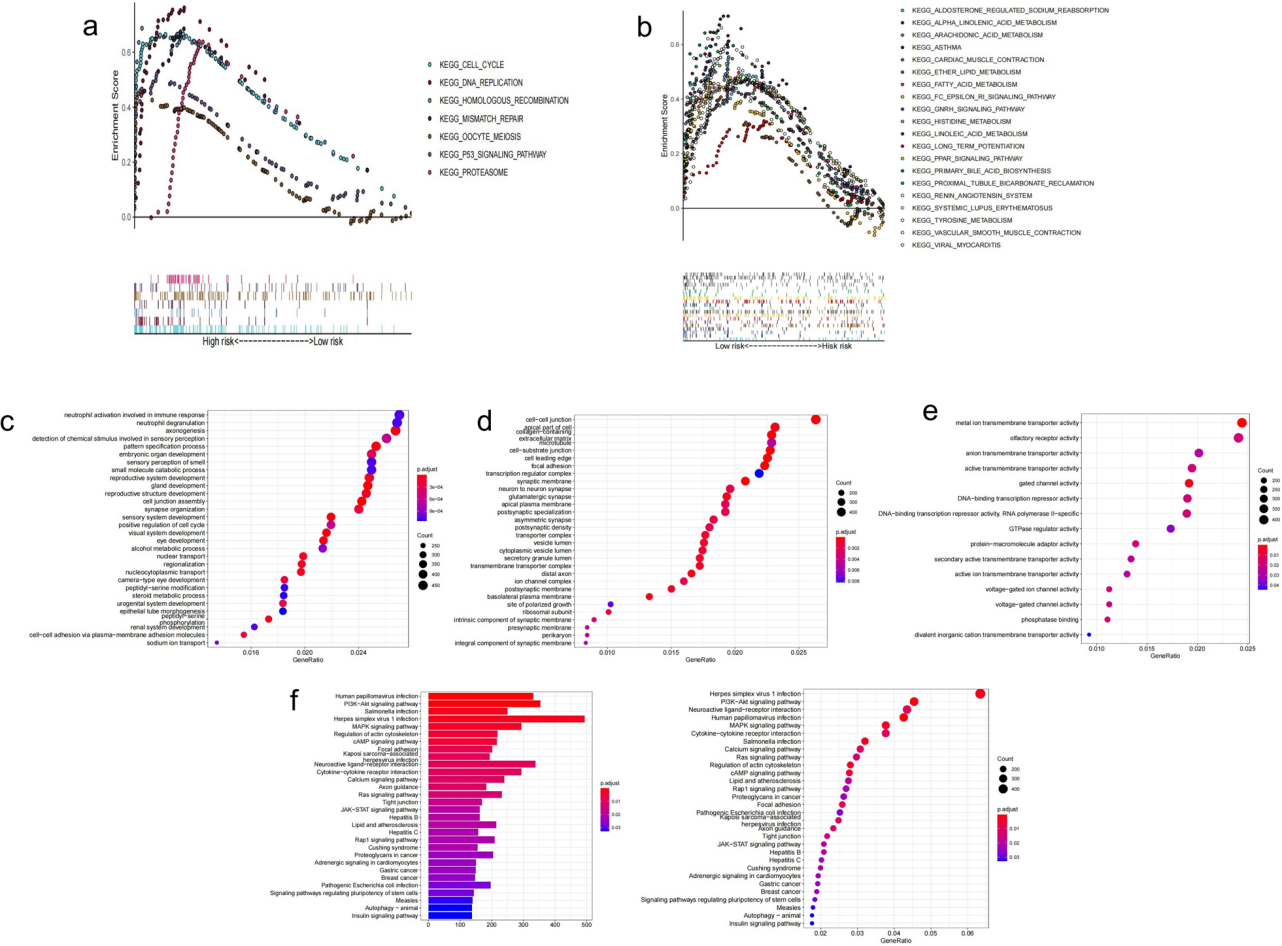
The enrichment of the IRLPs subgroups and different expression gene set between IRLPs subgroups **a** KEGG analysis of high-risk IRLPs subgroup, **b** KEGG analysis of low-risk IRLPs subgroup, **c** GO-BP analysis of different expression gene set between IRLPs subgroups, **d** GO-CC analysis of different expression gene set between IRLPs subgroups, **e** GO-MF analysis of different expression gene set between IRLPs subgroups, **f** KEGG analysis of different expression gene set between IRLPs subgroups

**Fig. 10:**
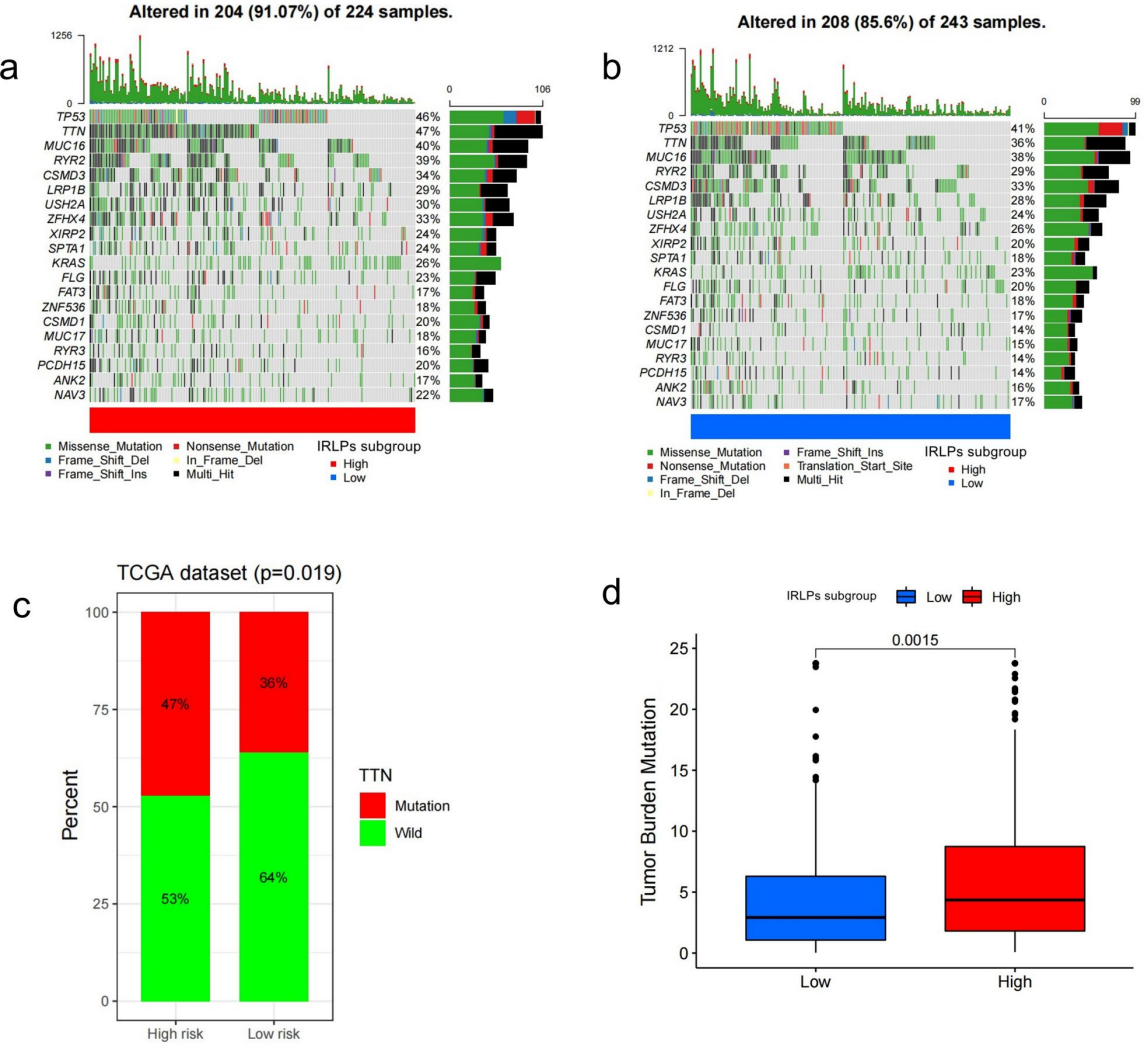
Gene mutations of different IRLPs subgroups **a** Top 20 genes mutation of high-risk IRLPs subgroup, **b** Top 20 genes mutation of low-risk IRLPs subgroup, **c** relationship between TTN mutation and IRLPs subgroups, **d** relationship between TMB and IRLPs subgroups

The gene sets of the high-IRLP subgroup were most enriched in KEGG_DNA_REPLICATION (enrich score = 0.76), GOBP_ATTACHMENT_OF_MITOTIC_SPINDLE_MICROTUBULES_TO_KINETOCHORE (enrich score = 0.92), GOMF_SINGLE_STRANDED_DNA_HELICASE_ACTIVITY (enrich score = 0.86), and GOCC_CONDENSED_NUCLEAR_CHROMOSOME_KINETOCHORE (enrich score = 0.83) (Fig. 9a). The gene sets of low-IRLPs subtype were most enriched in KEGG_ASTHMA (enrich score = 0.70), GOMF_ATP_DEPENDENT_MICROTUBULE_MOTOR_ACTIVITY_MINUS_END_DIRECTED (enrich score = 0.81), GOCC_AXONEMAL_DYNEIN_COMPLEX (enrich score = 0.79), and GOBP_SODIUM_ION_EXPORT_ACROSS_PLASMA_MEMBRANE (enrich score = 0.76) (Fig. 9b). GO analysis showed that the differentially expressed genes between the IRLP subgroups were enriched in neutrophil activation involved in immune response (BP), the cell–cell junction (CC), and metal ion transmembrane transporter activity (MF; Fig. 9c–e). KEGG analysis showed that the differentially expressed genes were enriched in Herpes simplex virus 1 infection (Fig. 9f). Then, we analyzed gene mutations to gain further biological insight into the immunological nature of the IRLPs subgroups. The high-risk group had the highest mutation rate (the top 20 genes), and missense variations were the most common mutation type in the two subgroups (Fig. 10a, b). The TTN mutation was the highest in the high-risk group. Next, we explored the relationship between the TTN mutation and IRLP subgroups. The TTN mutation was significantly more frequent in the high-risk group (Fig. 10c). Finally, we compared the tumor mutation burden (TMB) between these two subgroups. As a result, TMB was higher in the IRLPs of the high-risk group (Fig. 10d).

### Prediction of drug sensitivity of immunosuppressors

**Fig. 11:**
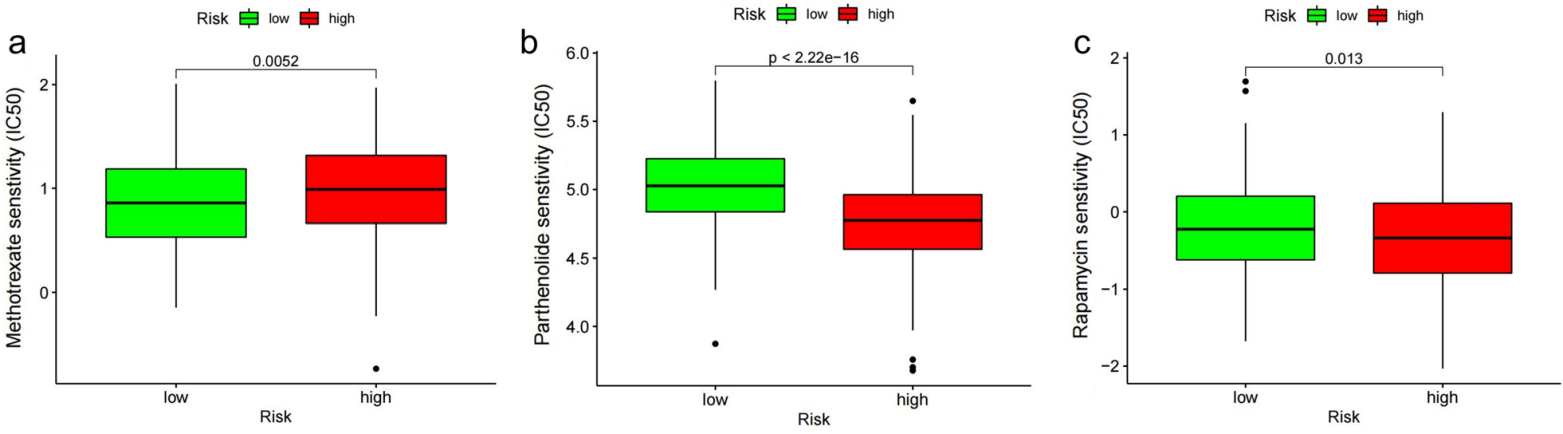
Prediction of drug sensitivity on immunosuppressors of IRLPs subgroups **a** methotrexate, **b** parthenolide, **c** rapamycin

We identified three immunosuppressors (methotrexate, parthenolide, and rapamycin) in the “pRRophetic” R package. Methotrexate had higher sensitivity in the IRLP high-risk group (*P* = 0.0052, Fig. 11a), whereas parthenolide (*P* < 0.001) and rapamycin (*P* = 0.013) showed lower sensitivity in IRLP high-risk group (Fig. 11b, c).

## Discussion

With the development of sequencing technology, more and more attention in cancer research has been paid to bioinformatics methods. In our present study, we constructed a risk scoring system based on eight IRLPs in the TCGA dataset, and the patients were divided into high-risk and low-risk groups according to the cut-off of risk score. Survival analysis showed that the high-risk group had a poor prognosis. The IRLP-risk score was an independent risk factor in our Cox regression analysis combined with clinical characteristics (age, gender, and stage). These results were also proven in the GEO dataset. Furthermore, our results also showed that the IRLP risk score was related to immune cell infiltration. Next, we explored the gene functional enrichment and gene mutation in two IRLPs subgroups. The high-risk group was found to be enriched in molecular changes in DNA and chromosomes, and to have a higher TMB than the low-risk group. Finally, the drug sensitivity of immunosuppressors was predicted to find the most suitable drugs for each group.

There are several studies developed prognostic signatures of LUAD based on RNA-seq or microarray expression in recent years [25, 26]. We built a prognostic IRLPs signature based on 8 IRLPs in our study, our results showed that patients with higher IRLPs risk score related to a worse prognosis and also could predict overall survival time (AUC = 0.777). What’s more, both internal and external queues were used to validate the IRLPs risk score system we built. All of the valid dataset indicated that our IRLPs signature was reliable (AUC_TCGA test dataset_ = 0.685, AUC_GEO dataset_ = 0.680). A previous study have built an immune-related prognostic signature directly with the expression level of genes based on RNA-seq for LUAD in TCGA dataset, the AUC for overall survival time is 0.662 [27], inferior to the AUCs in our study. The IRLPs risk score we constructed had a important prognostic significance, it may become a novel biomarker of LUAD.

The tumor microenvironment (TME) is correlated with cancer prognosis, supports cancer cells to replicative proliferation, and affects the malignant phenotypes [28, 29]. Many immune cells are present in the TME, modulating tumor cell migration, invasion, metastasis, and anticancer drug sensitivity [30]. The relationship between the IRLP score and infiltrating immune cells was analyzed in our study, and we found that they were significantly correlated. These results indicated that our IRLP risk score might allow the prognosis of LUAD by being sensitive to the functional status of immune cells. The immune score reflected the infiltration of immune cells in the tumor tissue based on the algorithm. A study found that patients with medium and high immune scores had a longer OS time than those in the low immune score group in lung cancer[31]. This means that a higher immune score may be beneficial for survival in lung cancer patients. The IRLP risk score was found to be negatively correlated with the immune score in our current results. These results demonstrated that a high immune activity might play an important role in the increased survival time of LUAD patients.

To gain further biological insight into the IRLP subgroups, we studied the functional enrichment and gene mutations in these two subgroups. Functional enrichment analysis found that molecular changes in DNA and chromosomes were most enriched in the high-risk subgroup. As previously reported, our results also showed that missense mutations are the most common type of mutations in LUAD[32]. The TTN mutation was found to be more frequent in the high-risk group than in the low-risk group and showed a significant difference between the high-risk and low-risk groups. The TTN mutation was reported as a potential biomarker associated with a better response to immune checkpoint blockade in solid tumors [33]. A study based on the TCGA dataset reported that the TTN missense mutation correlates with favorable prognosis in lung squamous cell carcinoma (LUSC) but not in LUAD [34]. Our results are also in agreement with the notion that TTN mutation plays a different role in LUAD.

Next, the relationship between the IRLP score and TMB was explored. Not only a high TMB was found to reflect worse clinical outcomes in non-small cell lung cancer [35], but also patients with high TMB (TMB-H) achieved good results in immunotherapy of solid tumors [36]. In this study, the high-risk subgroup had the higher TMB. Thus, the TMB may explain why IRLPs are correlated with the prognosis of LUAD, and the IRLP score may also help explain the immunotherapy response. However, other possible mechanisms involved in this relationship still need to be further studied.

With immune checkpoint inhibitors effectively improve the OS time in various cancers, immunotherapy-mediated ir AEs were frequently reported because of their specificity and severity [37, 38]. In the clinic, immunosuppressors can be used to treat severe ir AEs [39, 40] and play a relevant role as anticancer agents in recent decades [41, 42]. Since the immune systems of different individuals are not equally sensitive to drugs, immunosuppressors should further selected for LUAD therapy. Hence, we explored the drug sensitivity of three immunosuppressors: methotrexate, parthenolide, and rapamycin. Methotrexate usually used for autoimmune disease therapy, studies have reported that methotrexate has a good curative effect in rheumatic ir AEs [43–47], and fatal myositis can be successful cured by high-dose corticosteroids and methotrexate [48]. Our findings showed that irAEs in high-risk groups may be more sensitive to methotrexate treatment. Parthenolide is one of the biologicals that play an anti-inflammatory role by inhibiting nuclear factor kappa B (NF-κB) and cytokine tumor necrosis factor (TNF)-α [49, 50]. Parthenolide can induce lung cancer cells apoptosis and inhibit human lung cancer cell growth [51–53], demonstrated anticancer activity in the treatment of lung cancer. Rapamycin, an mTOR inhibitor, has broad anti-proliferative activity across NSCLC cells [54]. What’s more, studies have reported that combined application of rapamycin and other chemotherapy drugs would enhance the efficacy [55, 56]. In our exploration, the drug sensitivity of rapamycin and parthenolide were higher in low-risk group. These immunosuppressors (methotrexate, parthenolide, and rapamycin) have a different mechanism of action, and patients also had different drug sensitivities. Thus, our IRLPs scores may help identify the patients who would benefit from immunosuppressors therapy, but the mechanisms of drug action in these two subgroups still need to be clarified.

Although we have constructed an IRLP risk scoring system that showed a good predictive performance for LUAD patients and overcame the inconsistent sequencing platforms, there were still some noteworthy limitations. First, the patients included in the training set were downloaded from TCGA, which mainly includes white race patients. Thus, other ethnic groups still need to be evaluated. However, the results showed that the IRLP score constructed in TCGA also applies to the Asian GEO dataset. Second, we intersected the lncRNAs from two public datasets to overcome the differences in the sequencing platforms, and some important lncRNAs may have been ignored or contributed to selection bias. Third, our prediction on drug sensitivities was not validated in a cohort. There is no complete cohort data at present because of the difficulty in developing clinical immunosuppressive therapy experiment. Finally, we used the “pRRophetic” R package to explore the drug sensitivity of immunosuppressors, which includes a limited set of drugs and did not allow us to address the sensitivity of many commonly used drugs.

## Conclusion

In summary, we built a risk model based on IRLPs. This signature had a good predictive accuracy and effectiveness for LUAD. Furthermore, our IRLPs score significantly correlated with TME and TMB, indicating that these molecular changes might explain the different clinical outcomes. Importantly, our IRLPs may enhance the identification of the patients who can benefit from immunosuppressive therapy.

## Supplementary Information

**Additional file 1.** Methods in selection of 8 IRLPs.

## Data Availability

The original contributions presented in the study are included in the article/Supplementary Material. Further inquiries can be directed to the corresponding authors.

## Abbreviations

LUAD: Lung adenocarcinoma
LUSC: Lung squamous cell carcinoma
IRLP: Immune-related lncRNA pair
ir AEs: Immune-related adverse events
TCGA: The cancer genome atlas
GEO: Gene expression omnibus database
OS: Overall survival
TMB: Tumor mutation burden
lncRNAs: Long non-coding RNAs
KEGG: Kyoto encyclopedia of genes and genomes
GO: Gene ontology
GSEA: Gene set enrichment analysis
TME: Tumor microenvironment
