# Supplementary material for: Construction of an immune-related lncRNA signature pair for predicting oncologic outcomes and the sensitivity of immunosuppressor in treatment of lung adenocarcinoma
Source: Respir Res. 2022 May 13;23:123. doi: 10.1186/s12931-022-02043-4 (PMC9101821; doi:10.1186/s12931-022-02043-4)
Supplement: Supplementary file 1 — Additional file 1. Methods in selection of 8 IRLPs. [file 12931_2022_2043_MOESM1_ESM.doc]

Methods in selection of 8 IRLPs

Step 1: Immune-related gene set

2498 immune-related genes were downloaded from the ImmPort Portal (https://www.immport.org/)

Step 2: Identified immune-related lncRNAs

We performed Pearson’s correlation of mRNAs (immune-related genes) and lncRNAs, 105 immune-related lncRNAs (detect in TCGA platform and GPL570 platform) were selected in the TCGA dataset (|correlation coefficient| > 0.6 and *p* < 0.001).

Step 3: Constructed immune-related lncRNA pairs

All 105 immune-related lncRNAs were paired randomly to construct a collection of lncRNA pairs. For each LUAD sample, the IRLPs were computed by pairwise comparison of the expression level. The output is one if the expression of the first lncRNA is higher than that of the second one; otherwise, the output is zero.

Step 4: Construction of the immune-related lncRNA pairs signature

The TCGA dataset was randomly divide into train dataset and test dataset and we performed univariate Cox regression analysis and LASSO regression analysis with 10-fold cross-validation to find out OS-related immune-related lncRNA pairs in TCGA train dataset.

Finally, multivariate Cox regression analysis was carried out to identify top OS-related immune-related lncRNA pairs and to establish the final model of an immune-related lncRNA pairs risk score to predict the prognosis of LUAD.
